# Filling-Materials

**Published:** 1900-03

**Authors:** C. N. Johnson

**Affiliations:** Chicago, Ill.


### International Dental Journal.

Vol. XXI. MARCH, 1900. No. 3.

### Original Communications.[1]

### FILLING-MATERIALS.[2]

BY C. N. JOHNSON, L.D.S., D.D.S., CHICAGO, ILL.

A PROPER consideration of the filling-materials in use at the present time leads us at once to the conviction that we have no ideal material with which to fill teeth. We have materials which answer the purpose reasonably well under certain conditions, but no material which answers well under all conditions. It is therefore important that in the consideration of this question we study somewhat carefully the characteristics of the different materials and the indications for or against their use under the varying conditions found in the mouth. This must be done with the fact constantly in mind that no rigid or invariable rule may be laid down for the operator to follow in every case in the selection of his material. He must exercise his best judgment on the basis not only of expediency, but of the history of the various materials under long-continued service.

[1] The editor and publishers are not responsible for the views of authors of papers published in this department, nor for any claim to novelty, or otherwise, that may be made by them. No papers will be received for this department that have appeared in any other journal published in the ountry.

[2] Read before the American Academy of Dental Science, Boston, October 4, 1899.

### GOLD AND ITS COMBINATIONS.

Of all the materials yet introduced for filling teeth, gold must be acknowledged the peer. When properly understood and properly manipulated, under conditions favorable to its use, it is one of the most permanent materials we possess. It is exacting in its requirements, as are all things worthy, and he who would get the most from its use must adequately acquaint himself with its characteristics. These once understood, and the necessary skill developed to master the details of its manipulation, the operator is equipped with a material which is more reliable than any other, and more definite in results.

Its chief advantages consist in the fact that it may be made sufficiently hard to withstand the wear of mastication; that it is not acted on chemically by the fluids of the mouth so as to change color or disintegrate; that it remains stationary in form when properly condensed, and that it is uniform in its behavior when subjected to uniform methods of manipulation. This latter quality is really of much greater importance than a superficial consideration would suggest. It enables the operator to attain with it definite results, year after year. It will do to-day precisely what it did the day before, or what it did a year ago. This is not true of most other filling-materials, or at least, if it is true, the requirements for maintaining uniformity in the others are vastly more intricate and not so readily comprehended as with gold.

When it is stated that uniform results may always be obtained with gold, reference is made solely to its physical behavior. It is not intended to imply that teeth are uniformly saved by its use, even when it is manipulated to the best advantage. There are extraneous factors entering into the salvation or loss of filled teeth entirely apart from the intrinsic merits of the material with which they are filled, and gold cannot be exempted from these conditions. But it has a greater range of qualities entitling it to respect as a saver of teeth than any other one material, and its thorough understanding should be the aim of every practitioner.

Its disadvantages may be said to consist chiefly in the fact that it is somewhat exacting in its demands upon the operator; that it cannot be manipulated successfully under moisture; that its color renders it conspicuous for anterior teeth, particularly in individuals of certain types, and that it is a conductor of thermal changes. Another objection which must be considered in some patients is the length of time necessary for its insertion, with its corresponding tax on the individual, and its relative cost; though the fact should be strongly noted that a thorough mastery of the material by the operator will reduce much of this within the limits of tolerance.

Nor must the claim of its exacting nature be held in too high esteem as a disadvantage. This very requisite on the part of gold has done more than any other one thing in developing the skill of the dental profession to its present standard of excellence. Had it not been for gold, or, in other words, had all our filling-materials been of a plastic nature, dentistry never would have developed the brilliant manipulators who have graced its ranks. Gold is the stimulative astringent of the dental profession, keeping our operators keyed up to the highest point of proficiency by reason of its imperious demands upon their ability. A good gold-worker is enabled to perform all other kinds of dental service better as the result of his skill acquired in the manipulation of gold, and this sort of training has been the saving grace of dentistry.

Too many sins which belonged properly elsewhere have been laid at the door of gold. Men have attempted its use without a sufficiently developed skill, or without a proper understanding of its necessities. They have ignored its physical properties and its peculiar demands. Other men have essayed with it the impossible, and then attributed their failures to the material, thus laying gold unwittingly at fault.

The fact that gold cannot be successfully used under moisture is neither an unmixed evil nor altogether a disadvantage, when viewed in the light of the greatest perfection of results in our work. No filling, of whatever material, can be inserted under moisture as perfectly as if the cavity were dry, and this necessity of gold simply increases our care and leads to greater certainty of results. It has also made us more expert in maintaining dryness of teeth to be operated on.

The objection of color is a real one in many instances, and the vulgar display of gold in the mouths of the American people is greatly to be deplored. But this may largely be overcome, and the artistic sense of observers less seriously offended than it is without an abandonment of gold in the anterior teeth. A close study of the question will reveal the fact that gold is much more objectionable in some mouths than in others. In certain individuals a well-finished gold filling, beautifully polished without being burnished so as to glisten, is not at all conspicuous, even in an incisor, and not an offence to the æsthetic taste of the most exacting. In other individuals a gold filling in the anterior part of the mouth is at best an eyesore.

The difference in the effect of gold upon the appearance of individuals relates principally to the temperament and complexion of the patient, as well as to an æsthetic sense on the part of the operator, which may enable him to give his fillings artistic forms. The latter consideration should be carefully studied by every operator, to the end that gold fillings in the future should not be allowed to offend so glaringly as in the past, particularly in those instances where offence is not necessary. As to complexion, it will be found that decided blondes will tolerate gold in their anterior teeth with less objection than will brunettes. In fact, the color of gold harmonizes so well with the former that if the filling is well inserted there is nothing to offend the eye at a distance of several feet. On the other hand, a gold filling in the mouth of a brunette becomes at once conspicuous and objectionable. It is completely out of harmony with the features, and should never be tolerated except under circumstances of the most urgent necessity. This necessity seldom exists, in view of the fact that we have a material at hand which makes a filling scarcely discernible in these cases at a distance of ordinary conversation. This relates to a combination of gold and platinum which, under its proper head, will be considered in detail. The various gradations from brunette to blonde may be met with gold and platinum by using the different numbers as they come to us from the manufacturer, so that fillings may be made which will not be conspicuous, and every operator should acquaint himself with this material.

The question of thermal influence under gold fillings has claimed much attention from the profession, and there has been a large degree of misconception concerning it. Gold has been credited with more mischief in this particular than its merits warrant; for, while the material itself is a good conductor, it can be used in the mouth with little discomfort and little danger, provided proper precautions are taken. Gold is well tolerated, even in large cavities, if the pulp is not nearly exposed, or if there is not hypersensitiveness of the dentine. In the former case the pulp should be protected by an intermediate layer of cement before the gold is inserted, and in the latter case the hypersensitiveness should be controlled by medication previous to filling. Probably one of the best agents for this purpose is ninety-five per cent. carbolic acid.

One important factor connected with this question of thermal trouble relates to a condition apart from the filling itself. In the past, the profession has been very generally advised to leave in the bottom of cavities of any extent a portion of decalcified dentine as a protection to the pulp. Without going into the subject deeply enough to consider the character of this mass of tissue, it is safe to say that irritation is more often caused than avoided by it. It is more subject to impressions—thermal or otherwise—than is normal dentine, and a tooth, other things being equal, will respond to heat or cold more actively with decalcified dentine under a filling than if it had been thoroughly excavated to sound dentine. This cannot always be done short of pulp exposure, and pulp exposure should be avoided if possible; but the idea should ever be kept in mind that infected dentine is a menace to the future health and comfort of the tooth, and should be as thoroughly removed as may be.

If these precautions are taken, the trouble from thermal changes under a gold filling will be found for the most part temporary, and not of such serious import as has usually been attributed to it.

The indications for or against the use of gold in filling teeth relate to conditions most of which must be apparent to every observant operator. It should be used in all cases, if possible, where the greatest utility and the greatest permanence are expected of the operation. It should not be used where the conditions are such that it is manifestly impossible to accomplish perfect work with it. The control of the patient, whether young or old, is a necessary concomitant to the successful use of gold. It should not be attempted with a patient upon whom the physical or nervous tax would be too great, nor should it be employed in a tooth the peridental membrane of which is so greatly impaired as to revolt seriously against the impact of the mallet. In short, the best judgment and the closest discrimination should be exercised to the end that this king of all filling materials be not crucified by the enthusiastic unwisdom of its chief advocates.

### COMBINATIONS OF GOLD WITH OTHER MATERIALS.

*Gold and Platinum.*—This material makes a harder filling and one capable of greater wear than gold alone. It is also—as has been indicated—possible to produce with it fillings of varying degrees of shade which may be made to harmonize agreeably with the different types of patients which come under our hands. These degrees of shade are regulated by the percentage of gold and platinum in the given product. One preparation contains more gold than platinum, another about equal parts, while a third has a preponderance of platinum, and the color is thereby affected so as to range from a decidedly yellowish to a decidedly grayish tinge. This variation of the material may be used to striking advantage in harmonizing the filling with the features of the patient. The soft gray platinum shade falls in beautifully with the general effect in the mouth of a decided brunette, while the varying gradations from that to a light blonde may be followed with artistic results by a careful selection of the corresponding shades of material.

The combination of gold and platinum should be employed to a greater extent by the profession than it is to-day, for, while its manipulation is somewhat more exacting than that of gold, its intelligent use will lead to artistic results unattainable with gold alone, and its superior density adds greater permanence to the surfaces of all fillings which are in any way subject to attrition.

*Gold and Tin.*—This combination of materials possesses qualities which should commend it to the favorable attention of the profession. If its limitations are understood and the cases carefully selected for its use, it will prove a source of great satisfaction both to patient and practitioner; and it is therefore worthy of sufficient merit to induce every operator to study its characteristics and master the details of its manipulation. The claim has been made that it possesses no virtues which may not be found in non-cohesive gold, but in two important particulars this would seem to be an error. The tin-foil imparts to the mass a quality which non-cohesive gold does not possess, viz., a lead-like consistency which makes the product tougher and more readily adapted to walls of cavities. A plugger-point will not penetrate gold and tin so easily as it will a similar mass of non-cohesive gold; and another important item is the fact that the filling will build up more rapidly under the plugger than will gold with equal manipulation. A filling of gold and tin may therefore be inserted in less time than a similarly condensed filling of gold. But probably the most important difference between this combination and non-cohesive gold lies in the fact that in most instances, after a filling of gold and tin has been inserted for a time, the material undergoes a change which renders it much harder than it originally was, or than non-cohesive gold can possibly be made. It becomes crystalline in character, so that the filling is an integral mass, with little distinction between the gold and the tin. When it is first inserted, it is easily picked apart; but after several years' service in the mouth it becomes almost vitreous in nature, so that an excavator when drawn across it will respond with a metallic vibration. It has lost its dead softness and taken on a crystalline character which greatly increases its resisting properties and adds to its serviceability.

Its limitations consist in the fact that it will discolor in the mouth so that it cannot be used in any position where it may be seen, and also that it can never be built into contours or used in cavities of sufficiently large area to bring any considerable attrition of mastication upon it. The indications for its use relate principally to occlusal cavities in molars and bicuspids for children, and along the gingival third of deep occluso-proximal cavities in molars and bicuspids where the main body of the filling is to be of gold. It is especially useful in this latter case on account of materially shortening the operation and avoiding any possibility of discomfort from thermal changes, owing to the reduced conductive properties of the tin in the combination. The rapidity with which it may be inserted renders it a very desirable material in the mouths of children, where the avoidance of the rubber dam is an important consideration.

Gold and tin cannot be expected to do the same length of service as gold in any position where it is subjected to the constant attrition of mastication, and yet many of these occlusal fillings which have been under observation for ten or twelve years give every prospect of long-continued usefulness,—their length of service in most cases being out of all proportion to the limited time necessary for their insertion.

*Gold and Iridium.*—This combination has gained little attention from the profession, nor has it much to recommend it as a filling-material. By its use a harder surface may be given a filling than is possible with gold, but on being finished it presents a brassy appearance not pleasing to the eye, and it is therefore applicable only to posterior teeth. Even in these cases there is seldom an instance where gold and platinum will not do equal service and present a more artistic effect.

### AMALGAM.

This material has been at once the refuge and despair of the dental profession. It has probably saved teeth that never would have been saved without it, but, even in the hands of its most enthusiastic advocates, it has so often proved a disappointment that observant men can no longer remain blind to its limitations. The investigations of Fletcher, Flagg, Bogue, Black, Wedelstaedt and others have thrown much light on its characteristics; but even with the most that has been learned of it, and the best that has been said of it, the fact remains that much of the amalgam now offered the profession is illy adapted to the permanent saving of teeth. Nor are we likely soon to have in general use amalgams which may be uniformly depended upon,—not because a reasonably reliable grade of amalgam is impossible of manufacture, but because the conditions necessary to produce it are so exacting and the process so intricate that few men will be found sufficiently painstaking to invariably furnish it.

The chief faults with amalgam, as presented to us in the past, have exhibited themselves in a tendency to compress under the impact of mastication, so as to be drawn away from the cavity walls, but more particularly in a tendency to so change form, even after crystallization has taken place and where no undue pressure is exerted, as to produce a serious leak between the filling and the wall of the cavity. This is frequently exhibited in a decided crack along the cavity margins, easily visible to the naked eye, and capable of allowing the ingress of deleterious agents calculated to bring about recurrence of decay around the filling. These cracks do not need to be large enough to be seen in order to invite mischief, and very many teeth have been lost in the past as the result of this one characteristic of amalgam. The color of amalgam is also against it, but particularly the fact that much of the amalgam used by the profession has so changed color after its insertion in the mouth as to render it most unsightly. Neither has the blackening process always been confined to the material itself,—the teeth, in many instances, being so badly stained by it as to remain discolored for life.

These various faults of amalgam have claimed the attention of the profession for years, but no one would seem to have overcome them in any encouraging degree till the investigations of Dr. Black. After the most painstaking study of the physical character of the various alloys, he was finally enabled to produce one which would neither shrink nor expand, and which would sustain sufficient stress to make it reasonably serviceable in the mouth. But the conditions surrounding the manufacture and manipulation of such an alloy are so intricate and so exacting, and the ingredients so sensitive to the slightest variation in temperature or in treatment, that to produce a uniform product from one time to another would seem to be well-nigh beyond the possibility of human attainment. Manufacturers find that an ingot melted from a given formula may give a certain result, while another ingot from the same formula, and apparently treated in the same way, will show a variation in the result. The closest attention to the minutiæ is, therefore, necessary all along the line, from the refining of the original metals down to the filling and annealing of the finished product. Even then no one batch of alloy should ever be sent out short of a final test of the amalgam made from it by the most delicate machinery; and, in passing, it may be stated that when these tests are made they frequently prove a source of discouragement to the conscientious manufacturer. Discrepancies arise at every hand where, apparently, the greatest care had been taken with the preparation, and the more this amalgam question is studied the more it would seem to be hedged about by limitations so great as to be disheartening in view of the immense amount of the material being used at the present day. It would probably be better for the profession and the public if much of the energy which is now being expended on amalgam were diverted to other materials which are capable of more definite and uniform results.

And yet amalgam under existing conditions cannot well be excluded from our present list of filling-materials. It has been too useful for certain purposes, even with its limitations, to be entirely discarded. Its chief utility relates to the building up of teeth so badly broken down or so remotely situated in the mouth as to render the use of gold too exacting, and also to the saving of teeth whose peridental membranes are so impaired as to preclude the use of the mallet. Employed with discriminating care, amalgam may, under these conditions, serve a useful purpose; but it can never hope to attain to the same degree of excellence as a saver of teeth that has long since been established by gold.

### TIN.

The statement has often been made that this material does not claim from the profession the attention which its virtues merit, and this is probably true, though it would seem that the combination of gold and tin possesses all of the advantages of tin alone, together with the added virtue of being better able to resist wear on account of its greater hardness.

Tin may be used in one of two forms,—that of foil, or in the form of shavings cut from block tin. The former is perfectly non-cohesive, while the latter, if freshly cut, is said to possess cohesive properties, though tin cannot be built into contours with any assurance of permanence on account of its softness. The indications for the use of tin are practically the same as those suggested for gold and tin,—it being especially useful in any position where it is surrounded by four walls and is not subjected to wear. It is readily adapted to the cavity, will retain its form perfectly, except under pressure, and it is a poor conductor. This suggests that tin may serviceably be employed in simple cavities on all posterior teeth, such as buccal or lingual cavities of limited area, or in proximal cavities which do not involve the occlusal surface.

### CEMENTS.

There are three main varieties of cement,—the oxychloride of zinc, the oxyphosphate of zinc, and the oxyphosphate of copper. The *oxychloride of zinc* is indicated in pulpless teeth, for filling the pulp-chamber after the canals have been previously filled with gutta-percha, and also to form a lining to the cavity under the filling proper. It is seldom indicated in teeth with living pulps, particularly if there is a near approach to the pulp or if there is much hypersensitiveness, on account of its strong irritating properties. Neither can it be relied on for reasonable service in any position where it is subjected to the fluids of the mouth, from the fact that it is so readily dissolved,—this being especially true of proximal cavities at the gingival margin.

The *oxyphosphate of zinc* is an excellent agent as an intermediate under metal fillings in cases where there is a near approach to the pulp,—it being less of an irritant than the oxychloride,—and also for a temporary filling-material in the management of teeth which for any reason may not be in a condition for a permanent operation. Its chief limitation consists in a tendency to dissolve under the fluids of the mouth, though it is not so subject to this fault as is the oxychloride, and there is a considerable variation in its behavior in different mouths. In some instances it seems to wear well for years, particularly if the material used is of superior quality and it receives proper manipulation, but at best it may be accounted only a temporary expedient, and should not be relied on for permanent service.

The *oxyphosphate of copper*, introduced by Dr. Ames, of Chicago, is also somewhat soluble in the mouth, especially in vulnerable positions; and the fact that it is intensely black in color limits its use to positions not exposed to view. It is indicated in remote cavities on the necks of teeth occasioned by a recession of the gum, where the cavity is so ill defined as to make the use of gutta-percha or amalgam difficult. It may be made to adhere to the surface of a cavity very tenaciously, so that little undercutting is necessary, and it will prove an excellent expedient in that particular class of cases for which no other kind of filling seems suited.

### GUTTA-PERCHA.

This is a material which deserves more attention from the profession than it has received. In the particular field for which it is best suited it has no equal, and its uses are varied and unique in the saving of teeth. Its chief limitation lies in the fact that it is not sufficiently hard to withstand attrition, but placed in positions secure from wear it gives most excellent results. It is not dissolved by the fluids of the mouth, and it is one of the best of non-conductors. As a temporary sealing agent in the treatment of teeth it is, without question, the best material we possess. It is especially indicated for the filling of pulp-canals, being non-irritant, impervious to moisture, and readily molded to fit any inequality in the canal. It is also very valuable as a temporary filling-material in connection with oxyphosphate of zinc for proximal cavities, the gutta-percha being used in the cervical third of the cavity and the filling completed with cement. Gutta-percha will not dissolve out under these conditions, as will any of the cements; nor will the latter wear away so rapidly under attrition as will gutta-percha, so that by combining the two materials in this manner in the same cavity the operator gains the advantage of more adequate protection to the gingival margin and a better wearing service on the occlusal portion of the filling.

### INLAYS.

The discussion of filling-materials at the present time would hardly be complete without a consideration of inlays. The desirability of controlling caries in the anterior part of the mouth, without the necessity for an objectionable display of gold, would seem to be apparent, as also would the possibility of saving badly decayed teeth in any location where the insertion of gold is contraindicated on account of too great tax on the patient, or too much infliction on an impaired peridental membrane by the mallet. Crown-work, as the result of the considerations just indicated, has often been resorted to by operators in cases of extended decay at a period earlier than would make crowning justifiable if some more feasible means could be employed to tide the tooth over a period of several years. It is in cases of this kind that inlay work finds its most legitimate field. This would seem to be an intimation that inlays are to be considered more or less temporary in their nature, and from our past experience with them, and our present resources in their manufacture and methods of setting this fact, must seem undeniable.

It will scarcely do to point to a few isolated cases where inlays have done an excellent and, to all intents, a permanent service, and thereby claim that inlays are in the main trustworthy for extended use. There are principles involved in the process of their manufacture, and in the means employed to secure them in the cavity, which cannot by any sort of skill on the part of the operator, or any sort of reasoning on the part of their advocates, be overcome so as not to interfere with perfection of results. A metal matrix for receiving the material of the inlay, whether it be of gold, or glass, or porcelain, cannot by present methods be so adapted to the walls of the cavity as to prevent the penetration of moisture; neither can it be fashioned to remain firm in the cavity without something to hold it. To overcome these mechanical discrepancies, some material must be used to seal the interstice between the inlay and the cavity, and to retain the inlay in position by reason of its adhesive properties. Up to the present time no suitable material has been discovered which is not open to the objection of being more or less soluble in the fluids of the mouth. It is true that with an expert and careful operator, and a material manufactured and used in the light of the most recent investigations, these imperfections may in some degree be overcome, but with our present knowledge they cannot hope to be entirely obliterated.

If the too enthusiastic advocates of inlays would recognize this fact, and would more carefully discriminate in their selection of cases, the future of inlay work would be more secure than it seems to be at present. There is a useful purpose for it to perform in the profession, but from existing indications it would appear that it is doomed, as many another worthy object, to be made the victim of its over-zealous friends.

The cases most suited to the reception of inlays are in cavities on exposed surfaces of the anterior teeth, and in large cavities in bicuspids and molars where filling operations would prove too exhausting. The former should be of porcelain, for æsthetic reasons, while the latter should be of gold Gold inlays are more easily made than porcelain, and in localities subject to the stress of mastication are much less liable to fracture. They should therefore invariably be used in any position not exposed to view.

It should be a cardinal principle with every operator who uses inlays to so inform his patients on the subject that they shall return at stated intervals for examination of the work, with the expectancy of an occasional resetting as the result of disintegration of the cement. To do less than this with our present light on the subject is to be seriously derelict in duty.

A certain class of patients will cheerfully submit to the periodical replacement of porcelain inlays in preference to having an exhibition of gold in the mouth, and yet it may be well just at this point to look somewhat carefully into the probable results of doing too much of this temporary class of work. While some individuals possessed of a high sense of the æsthetic and with unlimited patience will follow up this work with the faithfulness its nature demands, the great majority will after a time become wearied and fall by the wayside. This is not conducive to confidence in the profession as to their ability to save teeth, and the general impression on the people will be unfortunate. The enviable reputation which dentistry possesses to-day as a calling capable of rendering unique and lasting service to the human race was not obtained by the performance of ephemeral operations. It is the result of hard, painstaking effort on the part of our illustrious predecessors, whose supreme effort was aimed in the direction of producing such work as would prove of the greatest possible permanence and utility to their patients. It is safe to conclude that it could never have been accomplished by the general use of inlay work.

